# 17β-Estradiol Promotes Apoptosis in Airway Smooth Muscle Cells Through CD38/SIRT1/p53 Pathway

**DOI:** 10.3389/fendo.2018.00770

**Published:** 2018-12-19

**Authors:** Yu Liu, Yinfang Guo, Weilu Huang, Ke-Yu Deng, Yisong Qian, Hong-Bo Xin

**Affiliations:** ^1^Cardiovascular Research Center, Institute of Translational Medicine, Nanchang University, Nanchang, China; ^2^Department of Medical Records, The First Affiliated Hospital of Nanchang University, Nanchang, China

**Keywords:** 17β-estradiol, CD38, SIRT1, hypoxia, apoptosis

## Abstract

17β-Estradiol (E2) is the major estrogen secreted by the premenopausal ovary and shows dual effects on cell apoptosis under pathological conditions. E2 was previously shown to increase CD38 mRNA and protein expression in myometrial smooth muscle, but its function and mechanism remain largely unknown. Here we investigated the role of E2 in hypoxia-induced apoptosis in mouse airway smooth muscle cells (ASMCs) and explored the underlying mechanisms. Results showed that E2 significantly increased CD38 expression at both mRNA and protein levels, accompanied with decreased SIRT1 levels in ASMCs. By using primary ASMCs from the wild type (WT) and the smooth muscle-specific CD38 knockout (CD38 KO) mice, we found that the down-regulation of SIRT1 induced by E2 was abolished in CD38 KO AMSCs. E2 promoted the acetylation of p53 in WT cells, and this effect was also diminished in the absence of CD38. In addition, E2 further activated CD38/SIRT1/p53 signal pathway and promoted cell apoptosis during hypoxia. However, these effects were reversed in CD38 KO ASMCs and by the specific SIRT1 activator Resveratrol. We also found that E2 enhanced CD38 expression through estrogen receptor. The data suggested that CD38 is a direct target for E2 which promotes hypoxia-induced AMSC apoptosis through SIRT1/p53 signal pathway.

## Introduction

Hypoxia is recognized as a critical contributor to pulmonary diseases including asthma, airway obstruction and pulmonary hypertension (1–3). Hypoxia stimulates airway inflammation and remodeling, and subsequently induces apoptosis in airway smooth muscle cells (ASMCs) during airway remodeling (4). There is emerging evidence for sex differences in the incidence and progression of lung diseases, and sex hormones play crucial roles in these pathological processes (5). Especially, estrogen regulates ASMCs in various manners. Estrogens reduce [Ca^2+^]i and promote human ASM relaxation via activation of cAMP and PKA, thereby facilitating bronchodilation (6). In addition, testosterone and E2 exhibit mitogenic effects in ASMCs, probably through estrogen receptors and the MAPK and PI3K signaling pathways, thus promote ASMC proliferation and airway remodeling (7). Estrogen signaling is also involved in allergic inflammation and contributes to sex differences in asthma and allergy (8). However, the effect of estrogen on ASMCs apoptosis during hypoxia remains largely unknown.

CD38 is a type II membrane-bound glycoprotein and functions as the major NADase responsible for the regulation of NAD-dependent deacetylase such as SIRT1 (9). In addition, CD38 is an NAADP synthase required for NAADP-mediated Ca^2+^ release from lysosomal stores (10). CD38/cyclic ADP-ribose (cADPR)-mediated calcium signaling plays critical roles in the regulation of intracellular calcium in a variety of smooth muscle cells, including that of the airway smooth muscle (11–13). Estrogens were shown to increase CD38 gene expression and leads to increased calcium mobilization and contractility of the myometrium (14, 15). It has also been recently reported that E2 downregulated SIRT1 expression in vascular smooth muscle cells, with increased apoptosis, reduced proliferation and migration, which were reversed by the SIRT1 activator Resveratrol (16). SIRT1 regulates p53-dependent apoptosis by deacetylating the Lys382 residue of p53, thus enhancing the transcriptional activity of p53 and inhibiting p53-induced apoptosis (17). However, whether E2 modulates the expression of CD38 and SIRT1 in ASMCs and the detailed mechanisms of E2 in the regulation of hypoxia-induced apoptosis have not been addressed.

In this study, we investigated the role of E2 in apoptosis during hypoxia by using primary ASMCs from the wild type (WT) and the smooth muscle-specific knockout of CD38 (CD38 KO) mice. CD38-mediated SIRT1/p53 signal pathway was also detected, with the purpose to elucidate the mechanism by which E2 promotes apoptosis in ASMCs.

## Materials and Methods

### Materials

E2 and Resveratrol were purchased from Sigma-Aldrich (St. Louis, MO). ICI182,780 was from Abcam (Cambridge, MA). The anti-CD38 antibody was obtained from R&D Systems, Inc. (Minneapolis, MN); the anti-SIRT1 antibody was from EMD Millipore Corp. (Temecula, CA); the anti-p53, anti-Acetyl-p53 (K379), anti-Bax and anti-Bcl-2 antibodies were from Cell Signaling Technology, Inc., (Danvers, MA), and the anti-glyceraldehyde phosphate dehydrogenase (GAPDH) antibody was obtained from KangChen Bio-tech Inc., (Shanghai, China).

### Preparation of Smooth Muscle-Specific CD38 Knockout Mice

Mice with LoxP flanking of exon 2 and exon 3 of the CD38 gene (CD38-fl/fl, produced by Cyagen Inc., Suzhou, China) were bred with mice expressing Cre recombinase under the control of a smooth muscle-specific promoter (SMA-Cre, from Collaborative Innovation Center of Model Animal, Wuhan University). The progeny with the genotype SMA-Cre-CD38-fl/fl is the homozygote used in the experiment.

### Isolation, Culture, and Characterization of ASMCs

Primary mouse ASMCs were prepared as previously described (18), with some modifications. Male, 8–10 weeks old WT or CD38 KO mice were anesthetized and the tracheas were aseptically excised and placed in Ca^2+^, Mg^2+^-free Hanks' balanced salt solution (HBSS). The isolated tracheas were cleaned of connective tissues, cut longitudinally through the cartilage, and enzymatically dissociated with HBSS containing elastase type I (2 mg/ml) and BSA (2.5 mg/ml) for 1 h in a water bath at 37°C. Dissociated cells in suspension were centrifuged and resuspended in Dulbecco's modified Eagle's medium (DMEM) supplemented with 10% fetal bovine serum (FBS), 100 U/ml penicillin, 100 μg/ml streptomycin, and 2.5 μg/ml amphotericin β. Cells were plated on culture flasks and grew until confluence at 37°C in humidified air containing 5% CO_2_. The confluent cells were passaged with 0.25% trypsin-0.02% EDTA solution. The cultures typically contained more than 98% ASMCs as assessed by immunocytochemical staining for the smooth muscle-specific marker α-actin. Cells at passages 3–5 were used for the experiments.

### Cell Culture Treatment and Hypoxia Exposure

For the concentration response assay, ASMCs were pre-treated with various concentrations of E2 (0.1, 1, 10, and 100 nM) for 24 h. For the time course assay, 10 or 100 nM of E2 were added to the cultures for 24 or 48 h incubation. In the subsequent experiments, WT and CD38 KO ASMCs were pre-treated with 10 nM of E2 for 48 h respectively, followed by the exposure of sustained hypoxia. Cells were maintained in a hypoxia chamber (1% O_2_, 5% CO_2_; balance N_2_ and water vapor) for 6 h to induce sustained hypoxia as described previously (19). A normoxic control experiment was performed in parallel by maintaining the cells under normoxia (21% O_2_, 5% CO_2_; 37°C). The specific SIRT1 activator Resveratrol (RSV, 10 μM) or the estrogen receptor antagonist ICI182,780 (ICI, 10 nM) was added to the cultures 2 h before E2 incubation.

### Real-Time PCR

Total RNA was isolated from ASMCs using the TRIzol™ reagent (Life Technologies, CA, USA) according to the manufacturer's instructions. One microgram of total RNA was reverse-transcribed using a One Step PrimeScript™ RT-PCR Kit (Takara, Dalian, China) with a thermocycler. Real-time PCR was performed using the ABI ViiA^TM^ 7 system with a reaction mixture that consisted of SYBR Green 2×PCR Master Mix (Applied Biosystems, CA, USA), cDNA template (0.5 μg), forward primer and reverse primer. Primer sequences were as follows: 5′-GAGCCTACCACGAAGCACTTTT-3′ and 5′-GGCCGGAGGATCTGAGTGTA-3′ (CD38), 5′-GCCAAACTTTGTTGTAACCCTGTA-3′ and 5′-TGGTGGCAACTCTGATAAATGAA-3′ (SIRT1), and 5′-ACATGGCCTCCAAGGAGTAAGAA-3′ and 5′-GGGATAGGGCCTCTCTTGCT-3′ (GAPDH). The PCR protocol consisted of 40 cycles of denaturation at 95°C for 15 s followed by 60°C for 1 min to allow extension and amplification of the target sequence. Data were analyzed using ABI 7500 sequence detection system software. The amount of mRNA was normalized to GAPDH using the 2^−ΔΔCT^ method. The results were from three independent experiments performed in triplicate.

### Western Blot

The cells were collected and lysed in RIPA lysis buffer. Equal amounts of protein per sample were loaded in each lane, separated by SDS-PAGE, and transferred to PVDF membranes. The membranes were blocked with skimmed milk for 1 h, washed in Tris buffered saline containing 0.1% Tween-20 (TBST) and incubated overnight with the primary antibodies. After washing three times with TBST, the membranes were incubated for 1 h at room temperature with horseradish peroxidase-conjugated goat anti-rabbit or anti-mouse IgG and donkey anti-sheep IgG. Bands were visualized using the SuperSignalWest Pico Chemiluminescent Substrate Trial Kit (Pierce, Rockford, IL, USA). Images were taken using the ChemiDoc XRS system with Quantity One software (Bio-Rad, Richmond, CA, USA).

### Hoechst 33258 Staining

Cell apoptosis was detected with DNA staining by Hoechst 33258. At the end of the treatment, cells were rinsed with phosphate-buffered saline (PBS, pH 7.4) and fixed with 4% paraformaldehyde for 30 min at room temperature, followed by incubation with Hoechst 33258 (5 μM, final concentration) at room temperature for 20 min. Fluorescence images were examined under the fluorescence microscope (Olympus IX71, Tokyo, Japan).

### Caspase-3 Activity Assay

Caspase-3 activity was measured in lysates of AMSCs using the CaspACE^TM^ Assay System, Colorimetric (Promega, Madison, WI) following the instructions of the manufacturer. Briefly, cells were lysed by freeze-thaw, and then incubated on ice for 20 min to ensure complete cell lysis. Cell lysates were centrifuged at 12 000 rpm for 10 min at 4°C, and the supernatant fraction was collected for the determination. An aliquot of culture supernatant was incubated with 200 mM of DEVDpNA substrate at 37°C for 4 h. The absorbance was measured at 405 nm. The luminescence was measured in a microplate reader and the protein levels in the lysates were determined by the method of Bradford. Results were expressed as a percentage of the control cells.

### Statistical Analysis

All values are expressed as the mean ± SD of at least three independent preparations. Differences among the groups were compared using one-way ANOVA analysis followed by a Tukey *post-hoc* test. A difference with *P* < 0.05 was considered statistically significant.

## Results

### E2 Increases CD38 Expression and Decreases SIRT1 Levels in ASMCs

**Figure 1:**
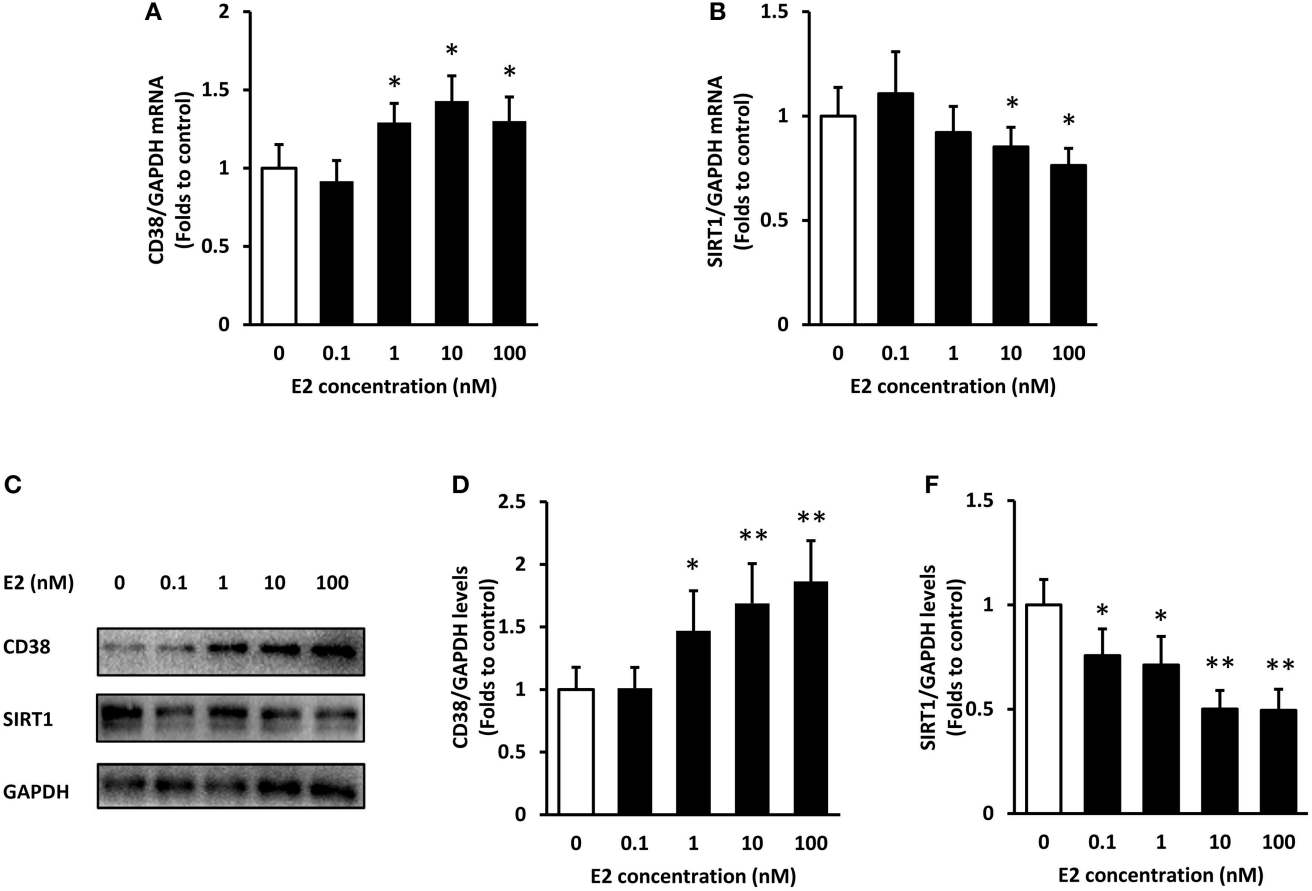
Expression of CD38 and SIRT1 in ASMCs after E2 treatment. ASMCs were pre-treated with the indicated concentrations of E2 for 24 h. **(A)** CD38 and **(B)** SIRT1 mRNA levels were detected by real-time PCR. **(C)** CD38 and SIRT1 protein levels were determined by western blot and quantitative analysis of **(D)** CD38 and **(E)** SIRT1 levels was normalized to GAPDH levels. *P < 0.05, **P < 0.01 vs. the control group. *N* = 3.

The expression of CD38 and SIRT1 at mRNA and protein levels were detected by real-time PCR and western blot, respectively. Non-normalized Ct values and non-cropped non contrasted western-blot images were provided in Supplementary Material. Firstly, ASMCs were pre-treated with various concentrations (0.1, 1, 10, and 100 nM) of E2 for 24 h. The mRNA levels of CD38 raised with the increase of E2 concentration, and the expression achieved maximum at 10 nM. There were no significant differences in CD38 expression between the 10 and 100 nM group (Figure 1A). By contrast, SIRT1 mRNA levels significantly decreased by the treatment of E2 at 10 and 100 nM (Figure 1B). In accordance with the PCR results, CD38 protein levels elevated whereas SIRT1 levels dropped in the presence of E2 in a concentration-dependent manner (Figures 1C–E).

**Figure 2:**
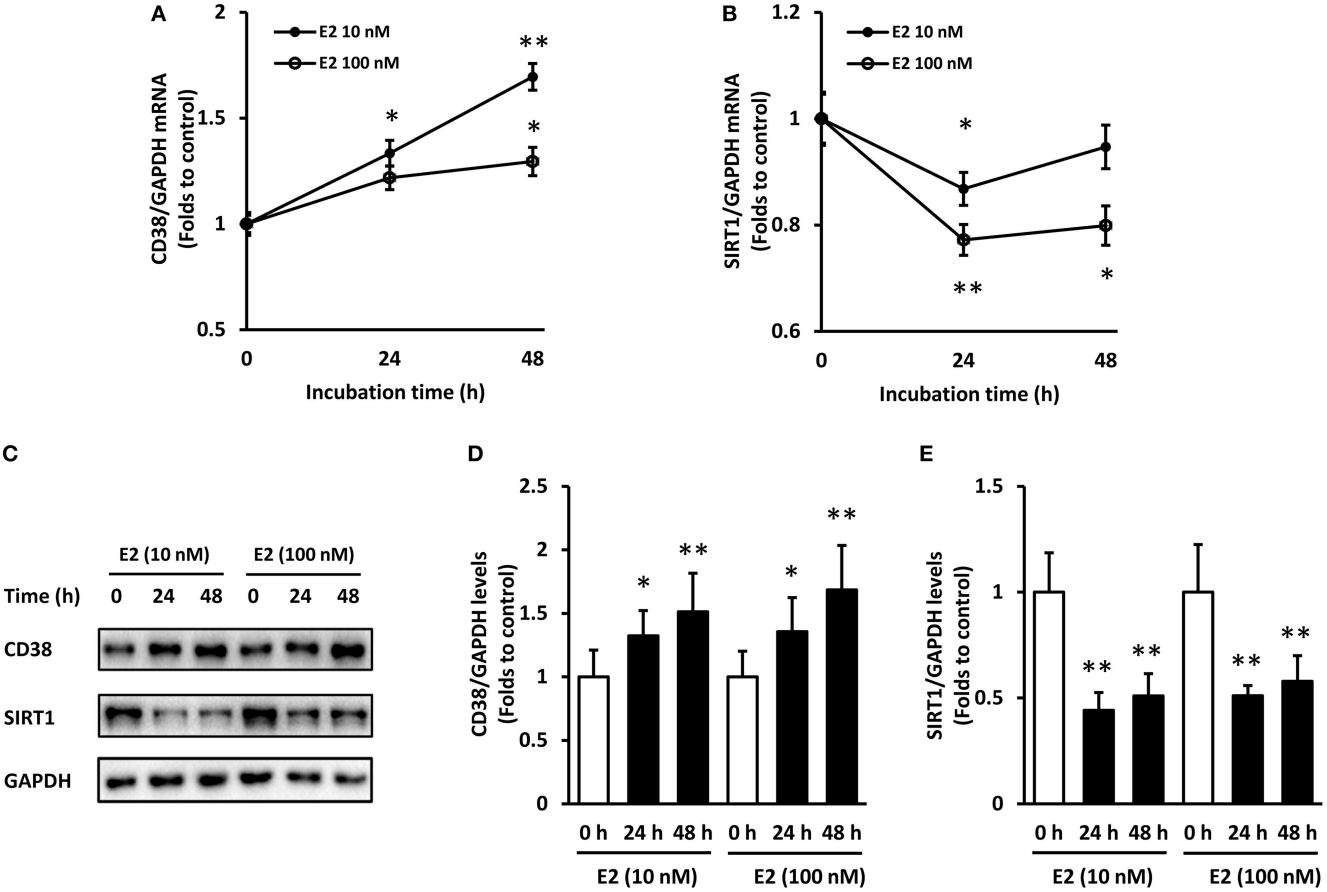
Time course of CD38 and SIRT1 expression in ASMCs with E2 treatment. ASMCs were pre-treated with 10 or 100 nM of E2 for 24 h and 48 h respectively. **(A)** CD38 and **(B)** SIRT1 mRNA levels were detected by real-time PCR. **(C)** CD38 and SIRT1 protein levels were determined by western blot and quantitative analysis of **(D)** CD38 and **(E)** SIRT1 levels was normalized to GAPDH levels. *P < 0.05, **P < 0.01 vs. the control group. *N* = 3.

In time course experiments, E2 at 10 and 100 nM were added to ASMCs for 24 and 48 h incubation, respectively. Results showed that CD38 mRNA continued to increase within 48 h and the effect was stronger in the 10 nM E2-treated group (Figure 2A). SIRT1 mRNA levels decreased at 24 h but partly restored at 48 h time point (Figure 2B). The protein levels of CD38 and SIRT1 showed a significant negative correction at 24 and 48 h, and there were no statistical differences between the 10 and 100 nM groups (Figures 2C–E). Therefore, pre-treatment with 10 nM of E2 for 48 h were selected for the subsequent experiments.

### E2 Acts on SIRT1/p53 Signaling Through CD38 in ASMCs

**Figure 3:**
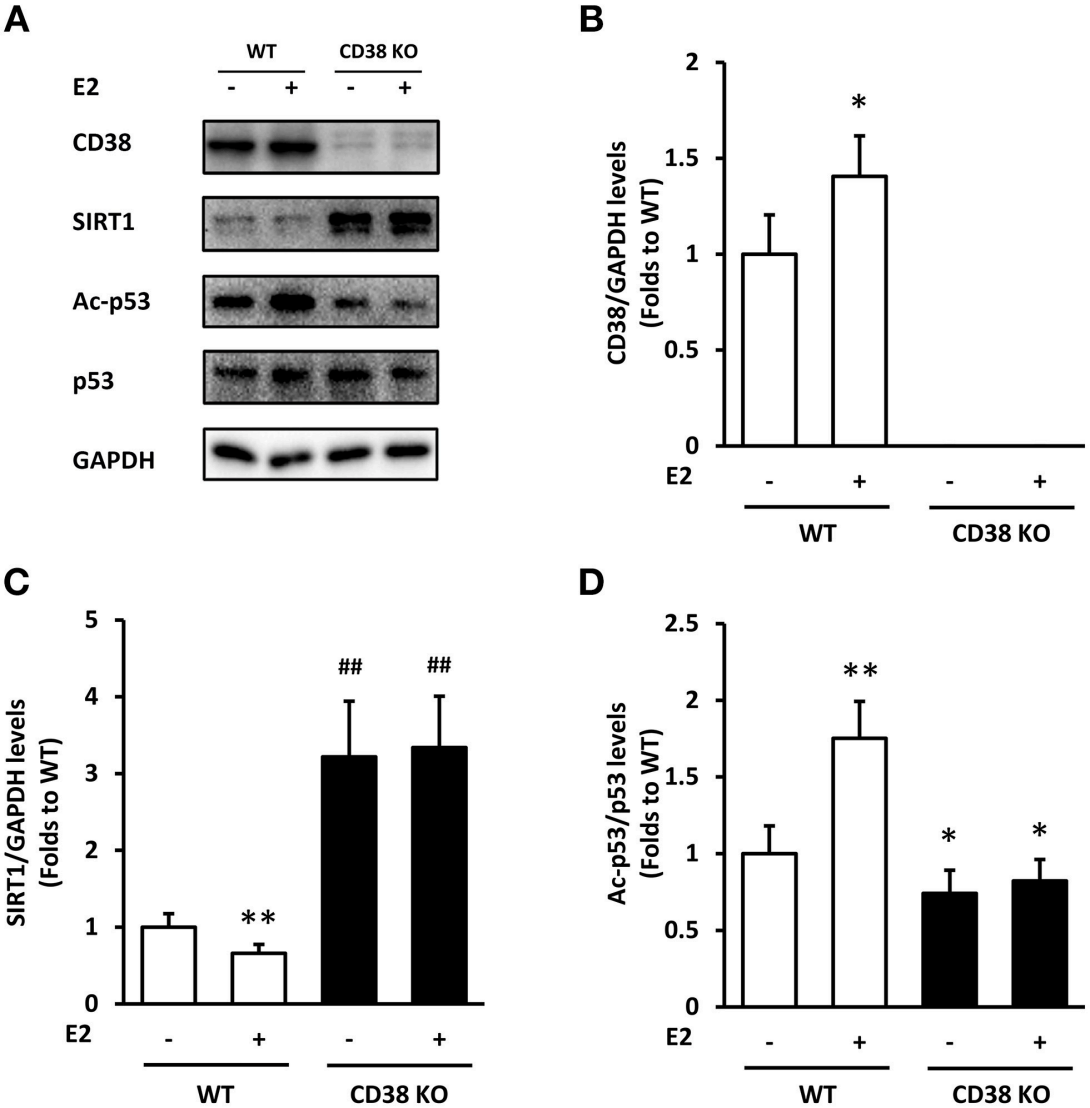
The effects of E2 on SIRT1/p53 signal pathway in WT and CD38 KO ASMCs. **(A)** CD38, SIRT1, p53, and Ac-p53 levels were determined by western blot. Quantitative analysis of **(B)** CD38 and **(C)** SIRT1 levels was normalized to GAPDH levels, and **(D)** Ac-p53 levels were normalized to total p53 levels. *P < 0.05, **P < 0.01 vs. the WT control group. ^*##*^*P* < 0.01 vs. the E2-treated WT group. *N* = 3.

We used the WT and CD38 KO ASMCs to confirm whether E2 affects SIRT1 expression through CD38. E2 promoted CD38 expression in WT ASMCs as expected (Figures 3A,B). The levels of SIRT1 were down-regulated by E2 in WT group compared with the vehicle treated cells. CD38 deficiency induced a marked increase in SIRT1 protein levels compared with the WT group, but this increase was not reversed by E2 treatment (Figures 3A,C).The acetylation of p53, one of the downstream targets of SIRT1, was assayed. In WT ASMCs, E2 increased the Ac-p53 levels, which were not changed in CD38 KO cells. The expression of p53 was not significantly altered (Figures 3A,D). These results indicated that E2 suppressed SIRT1/p53 signaling directly through CD38.

### CD38 Deficiency Reverses the Effect of E2 on SIRT1/p53 Pathway During Hypoxia

**Figure 4:**
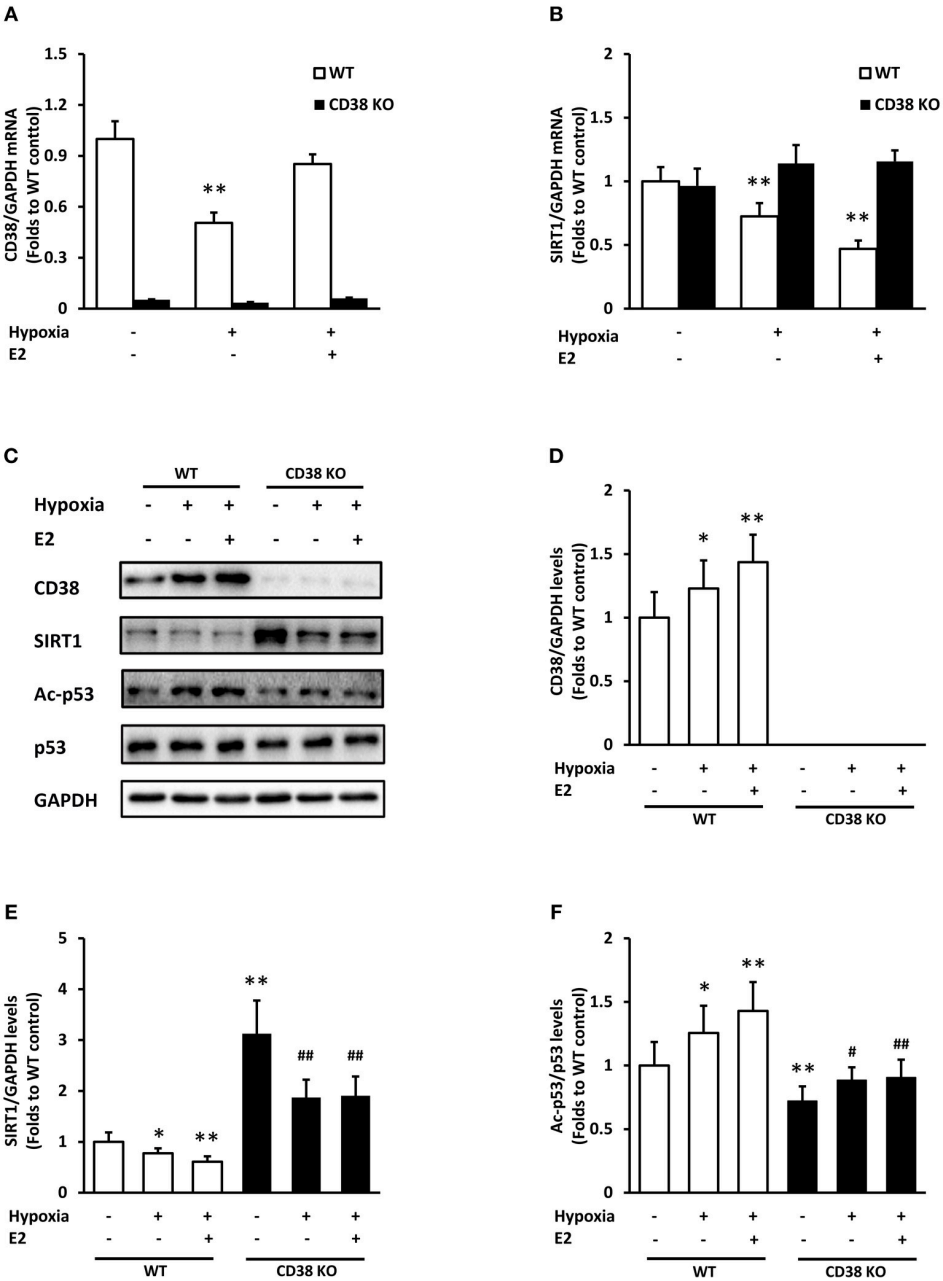
The effects of E2 on SIRT1/p53 signal pathway in WT and CD38 KO ASMCs after hypoxia exposure. **(A)** CD38 and **(B)** SIRT1 mRNA levels were detected by real-time PCR. **(C)** CD38, SIRT1, p53, and Ac-p53 levels were determined by western blot. Quantitative analysis of **(D)** CD38 and **(E)** SIRT1 levels was normalized to GAPDH levels and **(F)** Ac-p53 levels were normalized to total p53 levels. *P < 0.05, **P < 0.01 vs. the WT control group; ^#^*P* < 0.05, ^##^*P* < 0.01 vs. the corresponding WT group. *N* = 3.

We further investigated the role of E2 during hypoxia in WT and CD38 KO ASMCs. Hypoxia exposure induced an obvious down-regulation of CD38 mRNA, which was inhibited by E2 treatment in WT cells (Figure 4A). SIRT1 mRNA reduced after hypoxia, and E2 further suppressed its expression in WT ASMCs. However, the effect of E2 on SIRT1 expression was abolished in the absence of CD38 (Figure 4B). At protein levels, hypoxia resulted in an increase in CD38 and a decrease in SIRT1. E2 further promoted CD38 expression and suppressed SIRT1 levels. By contrast, the effect of E2 on SIRT1 disappeared in CD38 KO cells (Figures 4C–E). Hypoxia induced the acetylation of p53, which was also aggravated by E2 treatment. Accordingly, this effect was diminished in CD38 KO ASMCs (Figures 4C,F).

### CD38/SIRT1 Signaling Attenuates E2-Mediated ASMC Apoptosis After Hypoxia

**Figure 5:**
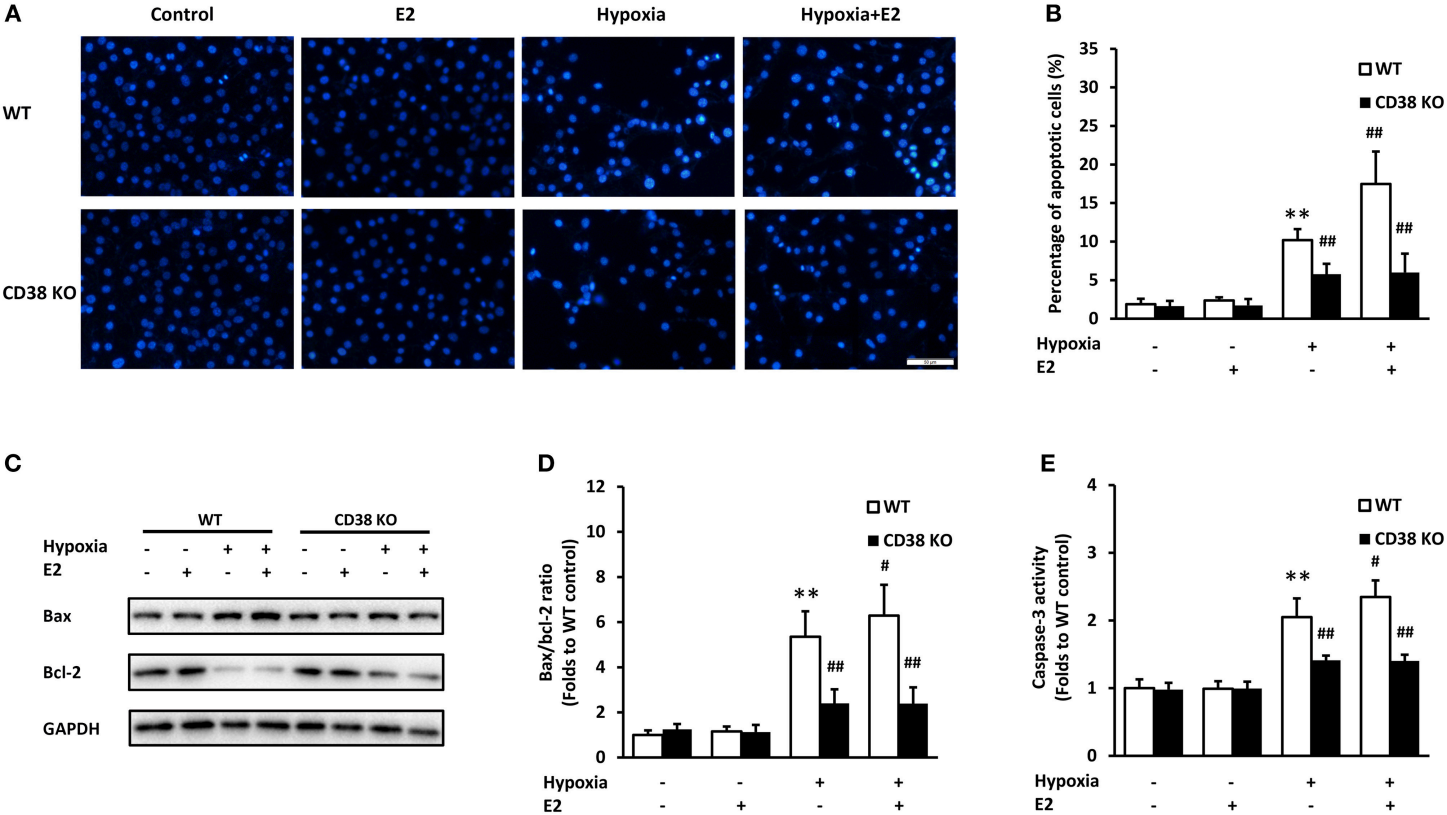
The effects of E2 on apoptosis in WT and CD38 KO ASMCs after hypoxia exposure. **(A)** Representative images of Hoechst 33258 staining in ASMCs. Scale bar, 50 μm. **(B)** Quantitative analysis of apoptosis expressed as the percentage of total cell count. **(C)** Bax and Bcl-2 levels were determined by western blot and **(D)** the Bax/Bcl-2 ratio were quantitatively analyzed. **(E)** The activity of caspase-3 was measured by colorimetry. **P < 0.01 vs. the WT control group; ^#^*P* < 0.05, ^*##*^*P* < 0.01 vs. the WT hypoxia group. *N* = 3.

We examined the apoptosis in ASMCs following hypoxia exposure. Hoechst 33258 staining was employed to evaluate the nuclear condensation and characteristic features of apoptotic cells. In WT ASMCs, control cells showed intact, light blue nuclei whereas cells exposed to hypoxia displayed typical nuclear apoptotic morphology, as indicated by bright, condensed and rounded nuclei. The apoptotic cells significantly increased after E2 treatment. In addition, E2 did not induce ASMC apoptosis under normoxia. However, CD38 deficiency showed an obvious protection against hypoxia exposure, with a marked reduction in apoptosis, and E2 did not further promote apoptosis in CD38 KO AMSCs (Figure 5A). The percentage of apoptotic cells was quantified in Figure 5B. Bax and Bcl-2 are the major members of Bcl-2 family which play a key role in promoting and inhibiting intrinsic apoptotic pathway. Bax promotes cell death while Bcl-2 prevents apoptosis by inhibiting the activity of Bax (20). The Bax/Bcl-2 ratio was significantly increased after hypoxia and E2 further aggravated the ratio. However, CD38 deficiency showed a lower Bax/Bcl-2 ratio compared with WT and the pro-apototic effect of E2 was antagonized in CD38 KO cells (Figures 5C,D). The effects of E2 on caspase-3 activation was further measured following hypoxia exposure. The activity of caspase-3 was comparable between the control and E2-treated cells under normoxia. Hypoxia induced a 2.05-fold increase in caspase-3 activity, and E2 further promoted caspase-3 activation in hypoxic WT AMSCs. However, in CD38 KO ASMCs, a lowed caspase-3 activity was observed after hypoxia exposure both in the absence and presence of E2 (Figure 5E).

**Figure 6:**
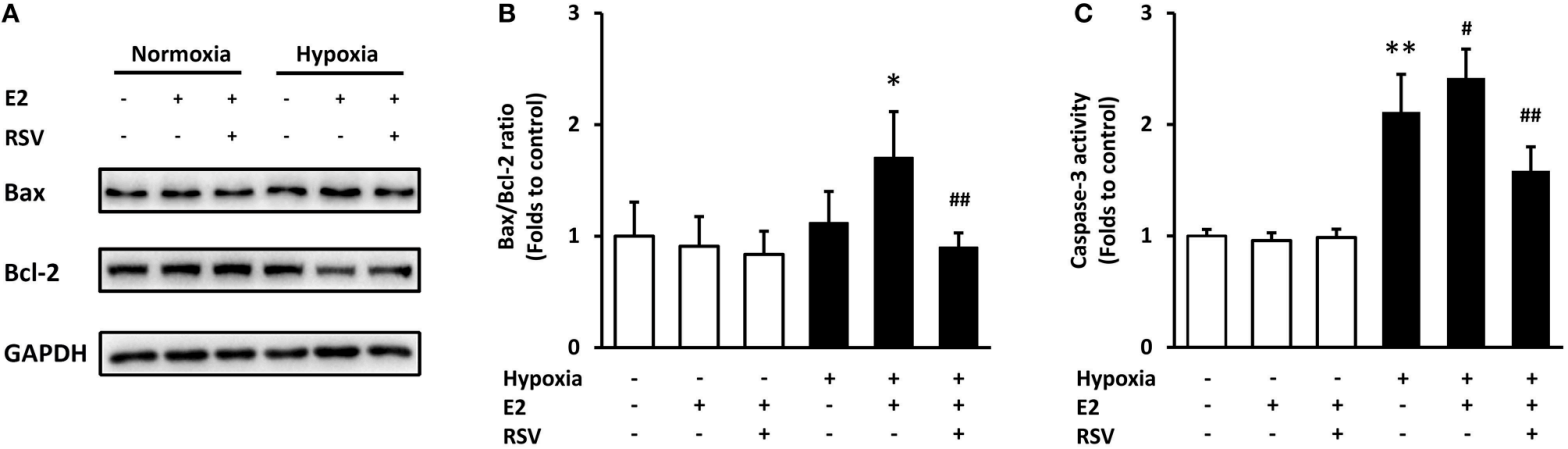
The effects of combined treatment with E2 and SIRT1 activator Resveratrol (RSV) on apoptosis after hypoxia exposure. RSV (10 μM) was added to ASMCs for 2 h incubation followed by 24 h of E2 treatment. **(A)** Bax and Bcl-2 levels were determined by western blot and **(B)** the Bax/Bcl-2 ratio were quantitatively analyzed. **(C)** The activity of caspase-3 was measured by colorimetry. *P < 0.05, **P < 0.01 vs. the control group; ^#^*P* < 0.05, ^##^*P* < 0.01 vs. the E2-treated group under hypoxia. *N* = 3.

The specific SIRT1 activator Resveratrol (RSV) was employed to verify whether SIRT1 is essential in E2-induced ASMC apoptosis. Single treatment with E2 or E2 combined with RSV did not affect apoptosis under normal conditions. However, E2 induced-Bax/Bcl-2 ratio change and caspase-3 activation were significantly reversed by RSV following hypoxia (Figure 6). The above data confirmed that E2 promoted ASMC apoptosis via CD38/SIRT1 signaling.

### E2 Enhanced CD38 Expression Through Estrogen Receptor

**Figure 7:**
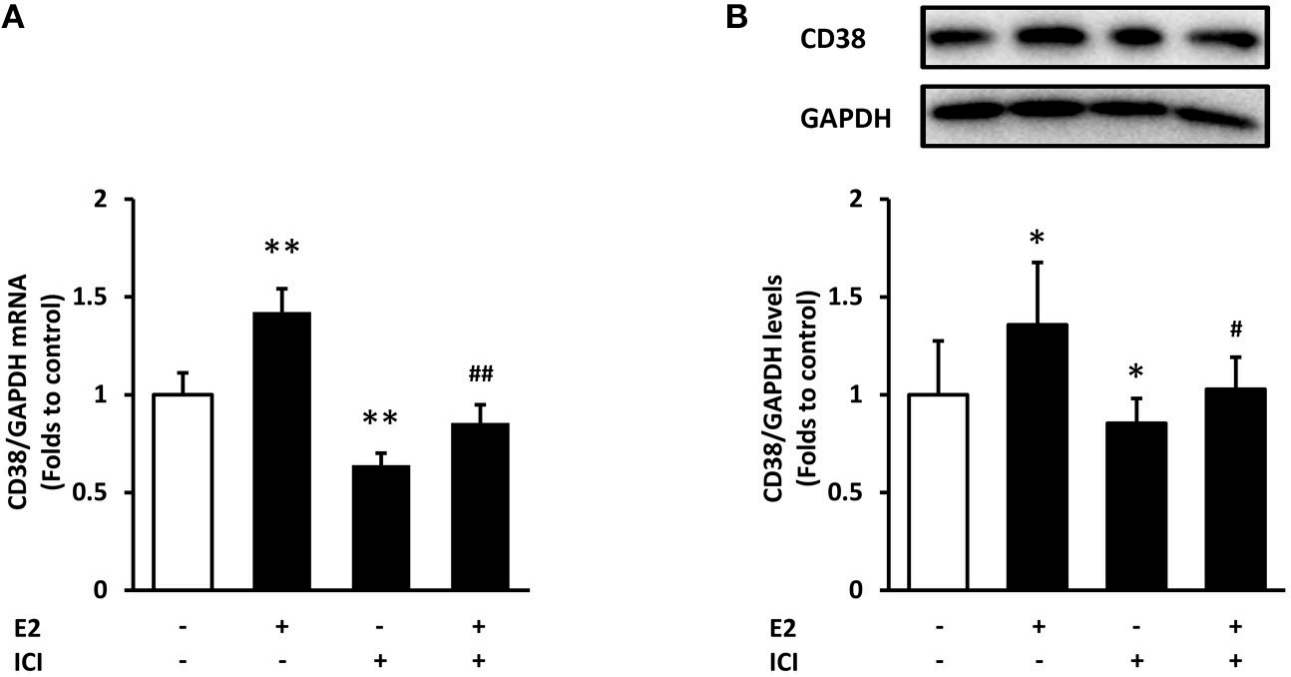
The effects of estrogen receptor antagonist on CD38 expression. The estrogen receptor antagonist ICI182,780 (ICI, 10 nM) was added to ASMCs for 2 h incubation followed by 24 h of E2 treatment. **(A)** CD38 mRNA levels were detected by real-time PCR. **(B)** CD38 protein levels were determined by western blot and quantitative analysis of CD38 levels was normalized to GAPDH levels. *P < 0.05, **P < 0.01 vs. the control group; ^#^*P* < 0.05, ^##^*P* < 0.01 vs. the E2-treated group. *N* = 3.

To verify whether estrogen receptors (ER) mediate the action of E2 on CD38 expression, we used compound ICI 182,780, an estrogen receptor antagonist with no partial agonist activity (21). Treatment with ICI182,780 significantly decreased CD38 mRNA and protein levels, and E2-indcued CD38 expression was completely abolished in the presence of ICI182,780 (Figure 7). This result suggested that E2 promotes CD38 expression through ER.

## Discussion

Here, we demonstrated that pretreatment with E2 significantly up-regulated CD38 expression and suppressed SIRT1 activation, thus increasing the acetylation of p53 in mouse ASMCs. E2 further exaggerated hypoxia-induced AMSC apoptosis while this effect disappeared in CD38 KO cells and in the presence of SIRT1 activator. By using the ER antagonist we also found that E2 enhanced CD38 expression through ER. These results suggested that E2 promotes apoptosis through CD38/SIRT1/p53 signaling pathway.

There is increasing evidence that sex differences exist in a variety of lung diseases including asthma and COPD, and sex steroids have complex effects in modulating the processes. For example, in adult women, the cyclical variations in sex steroid levels with the menstrual cycle may influence asthma symptoms. Worsening of symptoms usually occurs when estrogen levels reduce, suggesting that estrogens may be protective for asthma (22). However, Use of estrogen by hormone replacement therapy increases asthma symptoms and the risk of asthma onset (23). The confounding effects request much more research in modulation of asthma and other lung diseases to elucidate the mechanisms underlying sex differences.

Sex steroids modulate airway smooth muscle contractility in a variety of manners. Estrogens potentiate bronchodilation through prostaglandin synthesis and cGMP modulation, and further influence Ca^2+^ influx channels (24). The mechanism by which estrogens decrease Ca^2+^ responses probably involve ERα (25), inhibition of L-type channels and store-operated calcium channels (26). CD38 is a critical regulator for intracellular Ca^2+^ homeostasis. CD38 is capable of cleaving nicotinamide adenine dinucleotide (NAD) to cyclic ADP ribose (cADPR) which is a trigger for intracellular Ca^2+^ release and hydrolyzing cADPR to ADPR (27). In addition, CD38 is an NAADP synthase required for NAADP-mediated Ca^2+^ release from lysosomal stores (10). CD38 KO mice exhibit very low cADPR levels in the lungs, attenuated [Ca^2+^]i responses to spasmogens, and decreased airway responsiveness (28). Cytokines such as IL-13 or TNF-α caused significantly lower inflammation and hyperresponsiveness in the CD38 KO mice compared to WT controls (29, 30), suggesting the crucial roles of CD38 in the contractility of airway smooth muscle and airway hyperresponsiveness. Studies showed that E2 increased CD38 mRNA and protein expression, resulting in increased cADPR synthesis, which may contribute to calcium regulation and myometrial contractility in rat myometrium (31). However, there has no data for CD38 expression in airway smooth muscle. Therefore, we investigated the effect of E2 varying from 0.1 to 100 nM on CD38 mRNA and protein expression in ASMCs. In accordance with the result from myometrium, E2 showed a concentration-dependent increase in CD38 mRNA and protein levels. The time course assay revealed that CD38 mRNA and protein maintained a high level till 48 h with the treatment of physiological concentration of E2 (10 and 100 nM).

CD38 functions as the primary NAD^+^ hydrolase that maintains low intracellular NAD^+^ levels with a consequent low sirtuin activity (32) There is an increased NAD^+^ levels as well as SIRT1 enzymatic activity in CD38 knockout mice, which is responsible for the deacetylation of the SIRT1 substrate p53 (9). This non-genomic regulation may explain our current results that CD38 gene deletion markedly increases SIRT1 protein levels without significant effect on its mRNA expression. Several studies demonstrated the down-regulation of SIRT1 protein levels by E2 treatment in vascular smooth muscle cells (16, 33), but there was no data showing that E2 had effects on SIRT1 gene expression. In the present study, E2 induced obvious decrease in SIRT1 mRNA and protein levels in WT ASMCs but not in CD38 KO cells, suggesting that other unknown mechanisms may exist in E2's actions associated with CD38 and warrants further investigation in our feature work. Taken together, these results suggested that CD38 is necessary for the modulation of SIRT1/p53 signaling pathway by E2. We for the first time demonstrated that E2 modulates the CD38/SIRT1/p53 signal pathway in mouse AMSCs.

The SIRT1/p53 pathway mediated cell apoptosis in many pathological processes. It has been reported that high concentration of glucose results in neuronal apoptosis through downregulation of SIRT1 and increased acetylation of p53, which likely contribute to the development of cognitive impairment in diabetes (34). In another study, rotenone treatment promotes p53 transcription and apoptosis through targeting SIRT1 and H3K9 SH-SY5Y cells, leading to nigrostriatal degeneration in Parkinson's disease (35). Here we investigated whether E2 acts on CD38/SIRT1/p53 signal pathway during hypoxia in ASMCs. Results showed that hypoxia caused a marked decrease in CD38 mRNA levels, which is consisted with the previous study (36). However, CD38 protein levels was up-regulated after hypoxia exposure. Although both *in vivo* and *in vitro* studies have confirmed that CD38 is activated during the process of hypoxia or ischemia, triggering CD38-mediated NADP(H) depletion with loss of eNOS-mediated NO generation and increased eNOS uncoupling (37, 38), no studies showed the changes of CD38 protein levels under hypoxia. We speculated that different time course changes may exist in mRNA and protein expression following hypoxia, or there might be a negative feedback regulation that inhibits CD38 mRNA expression. E2 exacerbated hypoxia-induced SIRT1 suppression and p53 acetylation, and these effects were abolished in CD38 KO cells, suggesting that CD38 is an upstream signaling molecule that regulates hypoxia-induced SIRT1/p53 activation.

Bcl-2 and Bax are two main proteins of Bcl-2 family, which is notable for the regulation of cell apoptosis. Bcl-2, an anti-apoptotic protein, inhibits the accumulation of cytochrome c in the cytosol, thereby preventing caspase-3 activation and blocking the apoptotic cascade, whereas Bax was identified as the proapoptotic member that triggers the release of caspases. Therefore, the Bax/Bcl-2 ratio is a determining factor in the regulation of apoptotic cell death (39). The ratio of Bax to Bcl-2 increased following hypoxia exposure in different models (40, 41). The expression of Bcl-2 and Bax is regulated by p53. The activation of p53 induces the expression of bcl-2 while simultaneously stimulates the expression of bax (42). By measuring the fluorescence of Hoechst 33258, the Bax/Bcl-2 ratio and the activity of caspase-3, we demonstrated that E2 aggravated cell apoptosis following hypoxia stimulation. The application of CD38 KO cells and the SIRT1 activator further confirmed the direct role of CD38/SIRT1 in E2-mediated AMSC apoptosis.

Finally, we explored the underlying mechanism by which E2 modulates CD38 expression. It has been widely considered that estrogens perform physiological function through receptor-mediated signaling pathways. The nuclear ERs exist in two main isoforms termed ERα and ERβ, and the classical mechanism of estrogen action involves ligand-induced dimerization of ER which interacts with estrogen responsive elements (EREs) in target gene promoters and results in transcriptional activation (43). Here we determined whether E2 mediated CD38 transcription through ER, by using ICI 182,780. ICI 182,780 is a selective estrogen antagonist that has been used for assessing ER-mediated actions of estrogens (21). Results showed that ICI 182,780 suppressed CD38 mRNA levels and counteracted the effect of E2, indicating the involvement of ER in the regulation of CD38. However, much more work is needed to elucidate the molecular mechanism, including the identification of the isoform (ERα or ERβ) which mediates the effect, and exploring possible ERE on CD38 promoters.

According to our results, the physiological concentration of E2 affects CD38 expression and promotes apoptosis, indicating that E2 have adverse effects on ASMCs. This may probably explain why women are more susceptible to respiratory diseases and the clinical application of estrogens should be more cautious. Further research into the effects of estrogen on the proliferation and inflammatory response in ASMCs are necessary, and the animal models of specific pulmonary diseases such as asthma and pulmonary hypertension in the smooth muscle-specific CD38 KO mice will also provide essential tools for elucidating the function of E2 on ASMCs. These studies are now ongoing in our laboratory.

In summary, the estrogen E2 acts on CD38/SIRT1/p53 signal pathway, resulting the acetylation of p53 and pro-apoptotic effects in mouse ASMCS following hypoxia. The findings may provide novel evidence for the prevention and treatment of respiratory disease through CD38 inhibition.

## Ethics Statement

All experimental procedures involving animal and their care were carried out in accordance with the National Institutes of Health Guidelines. All experimental protocols were approved by Institutional Animal Care and use Committee of Nanchang University. All efforts were made to minimize animal suffering and reduce the number of animals used.

## Author Contributions

H-BX, K-YD, and YQ designed the experiments. The experimental procedures were performed by YL, YG, and WH. YQ and YL prepared the manuscript. H-BX revised the manuscript.

### Conflict of Interest Statement

The authors declare that the research was conducted in the absence of any commercial or financial relationships that could be construed as a potential conflict of interest.
