# Supplementary material for: 17β-Estradiol Promotes Apoptosis in Airway Smooth Muscle Cells Through CD38/SIRT1/p53 Pathway
Source: Front Endocrinol (Lausanne). 2018 Dec 19;9:770. doi: 10.3389/fendo.2018.00770 (PMC6305733; doi:10.3389/fendo.2018.00770)

Figure 1

CD38 (band 2-6)

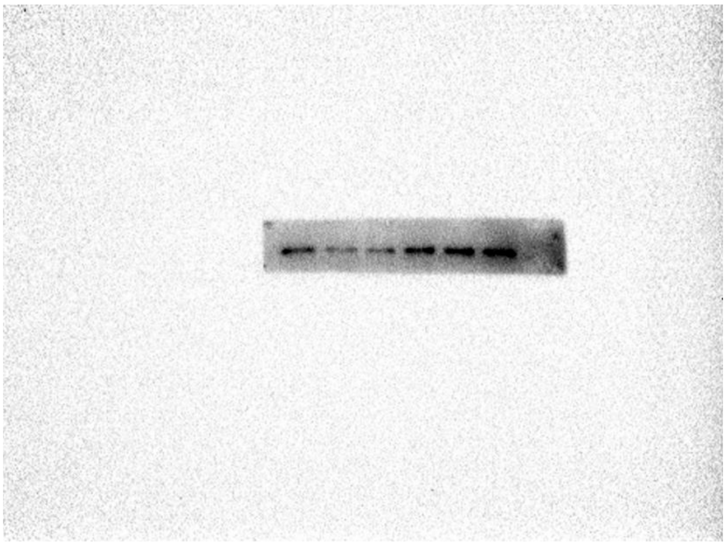

SIRT1 (band 2-6)

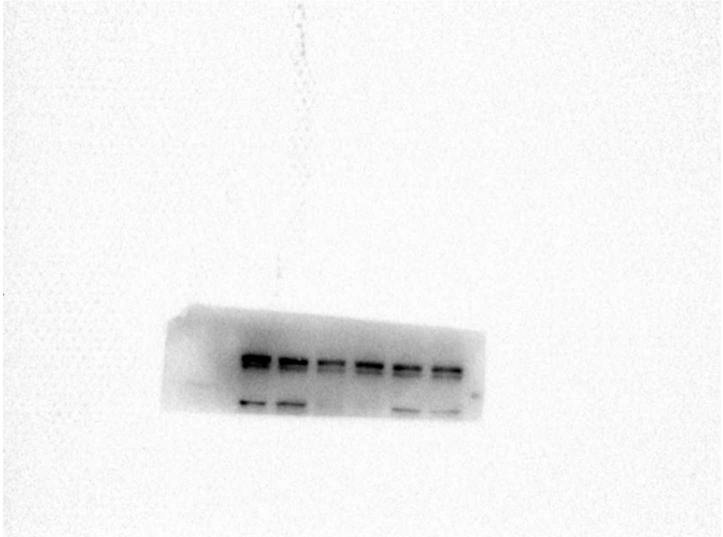

GAPDH (band 2-6)

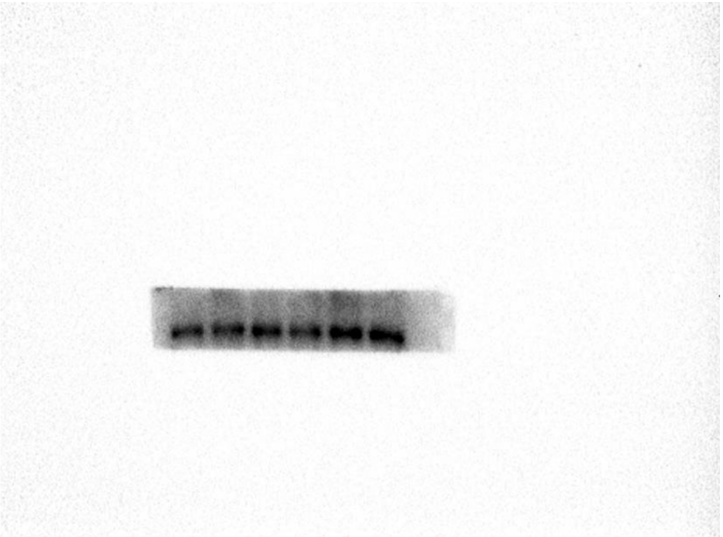

Figure 2

CD38

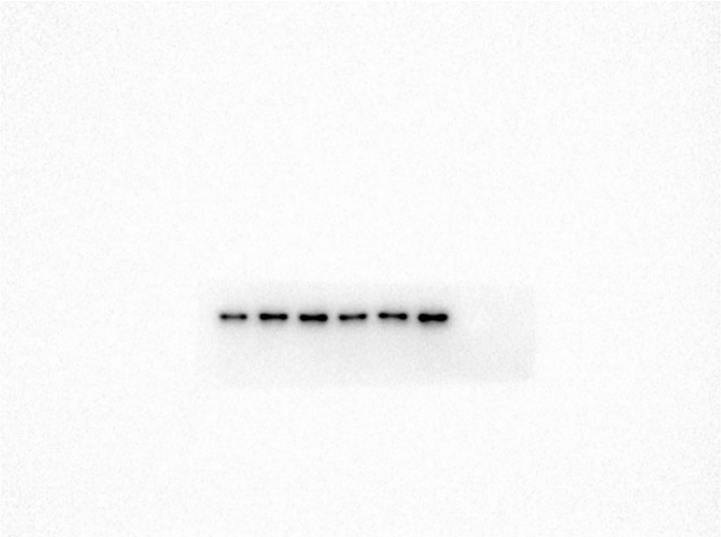

SIRT1

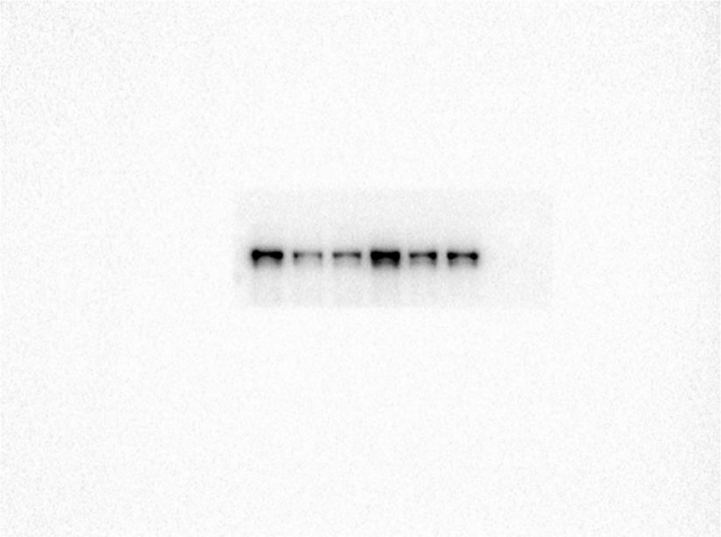

GAPDH

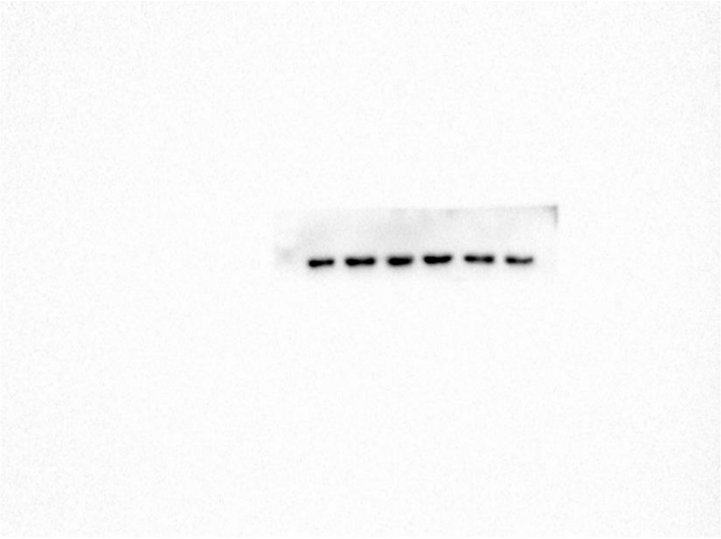

Figure 3

CD38

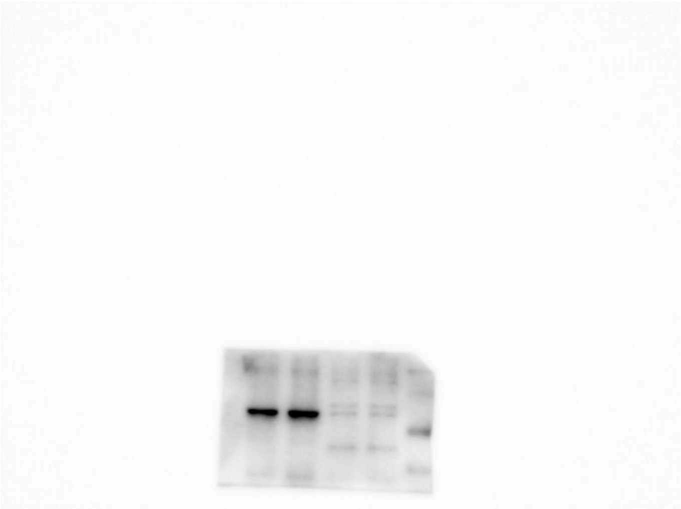

SIRT1

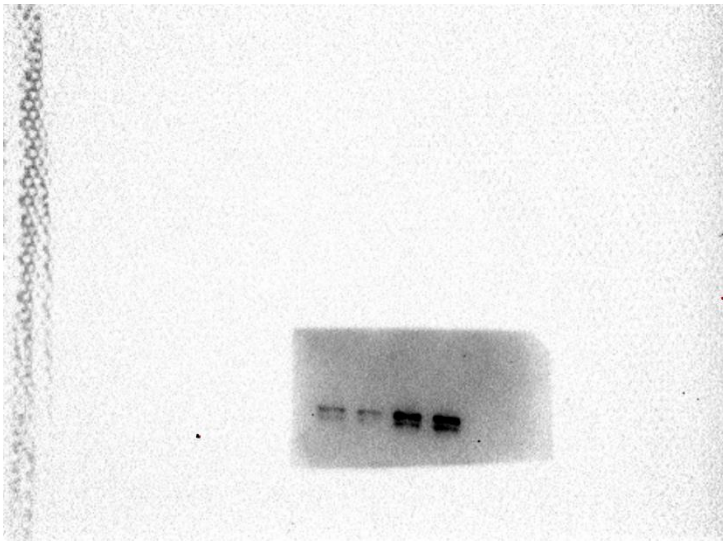

Acp53

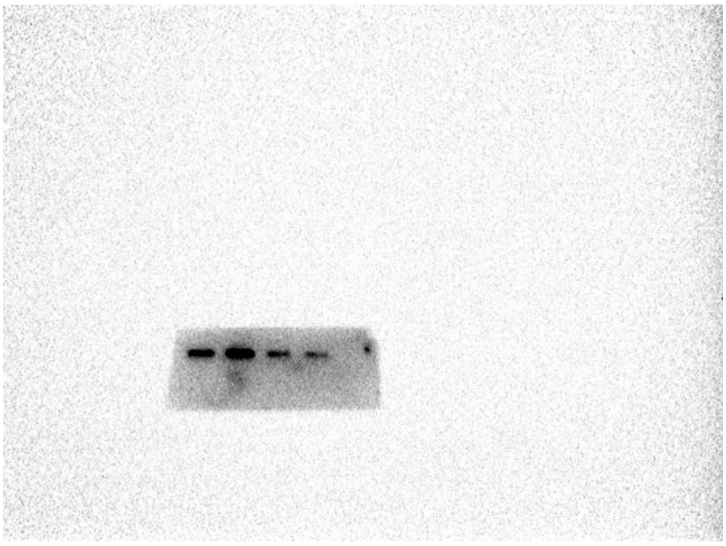

p53

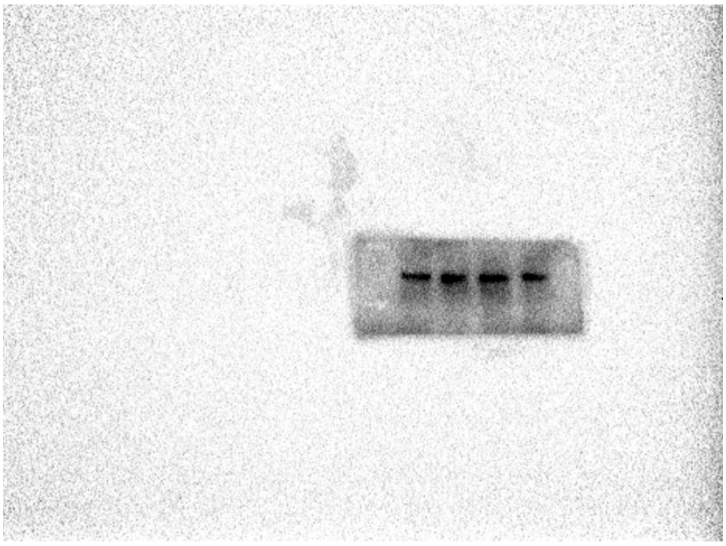

GAPDH

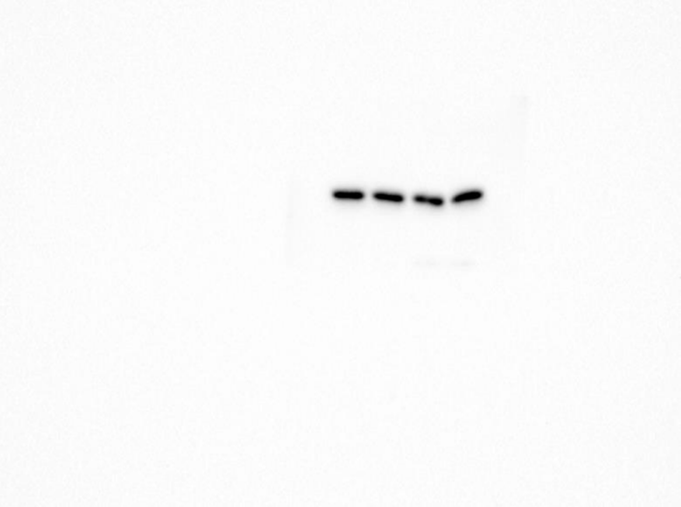

Figure 4

CD38 (left)

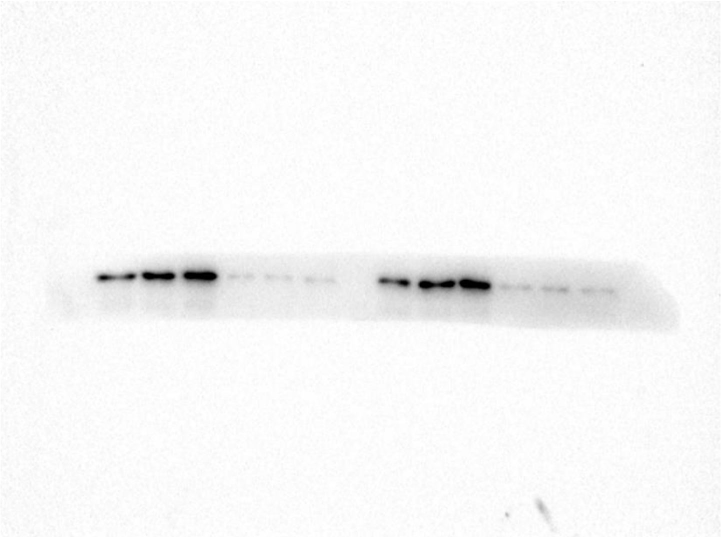

SIRT1

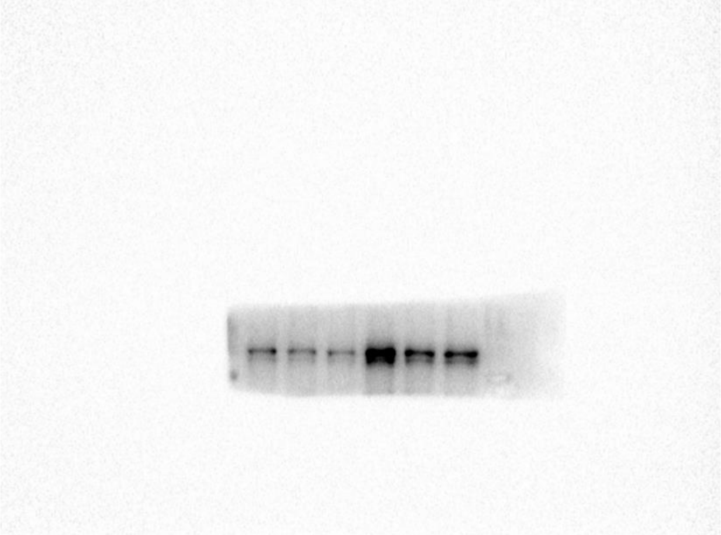

Acp53 (left)

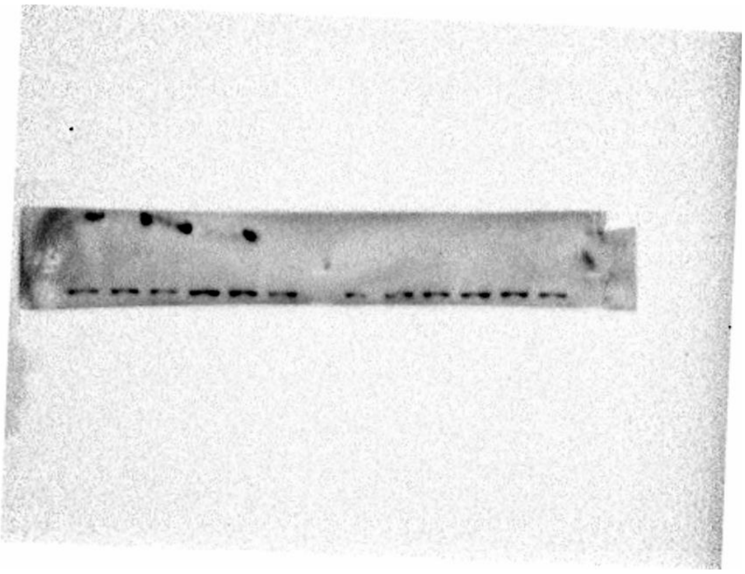

p53

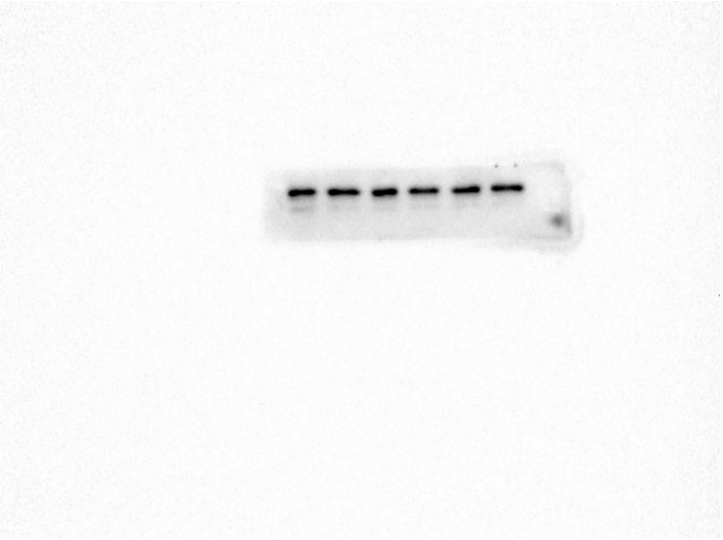

GAPDH

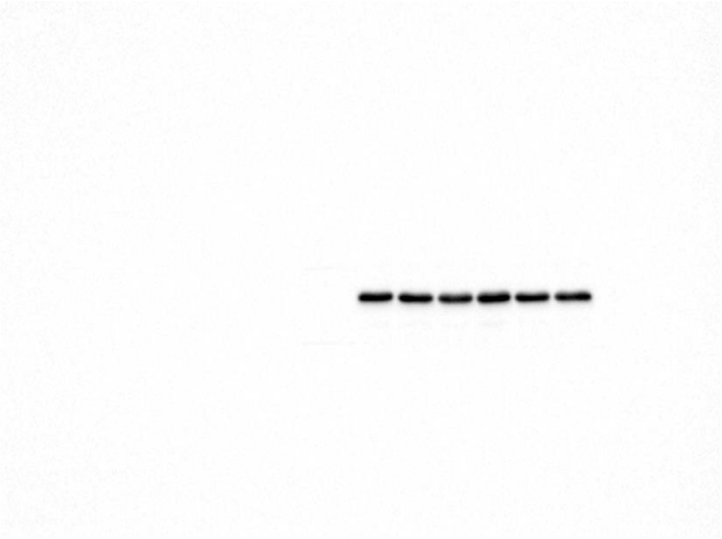

Figure 5

Bax

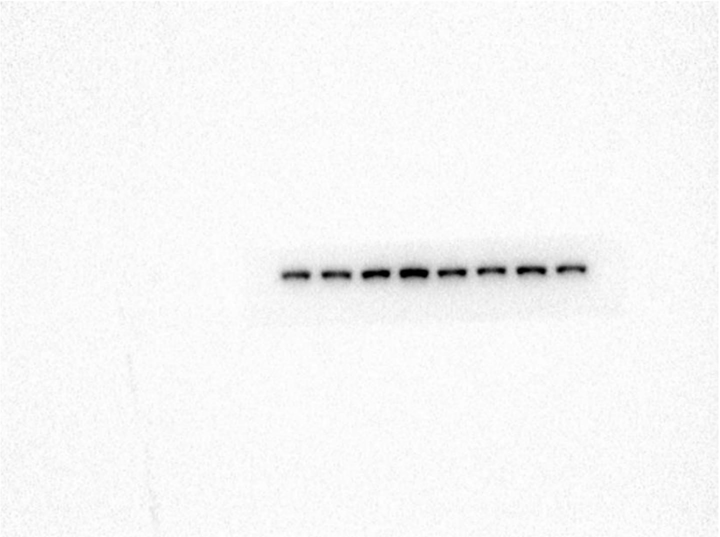

Bcl-2

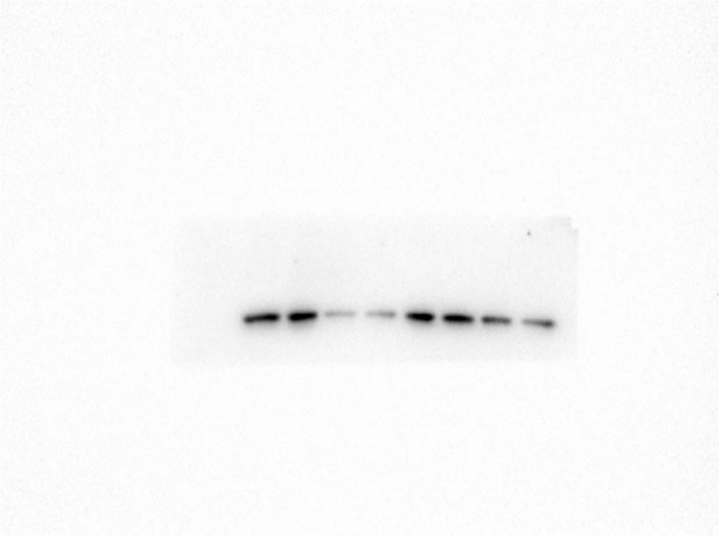

GAPDH

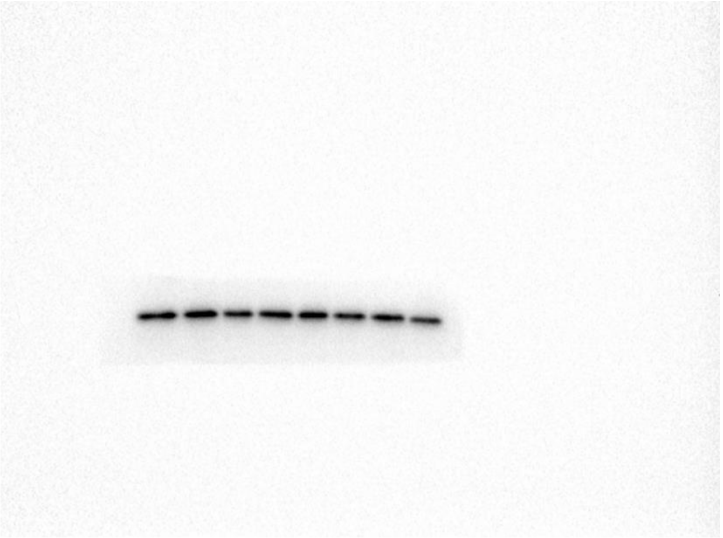

Figure 6

Bax

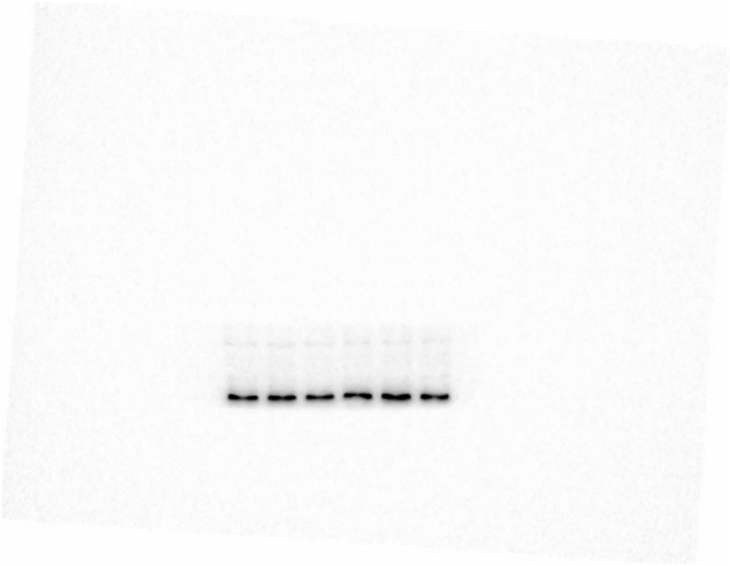

Bcl-2

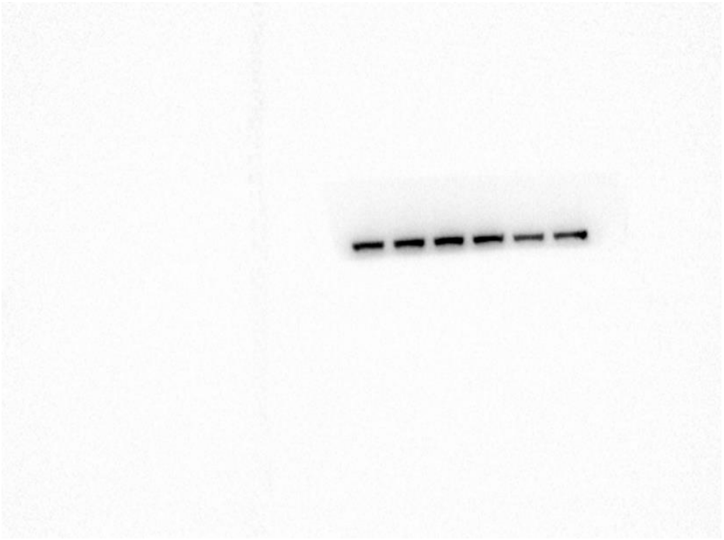

GAPDH

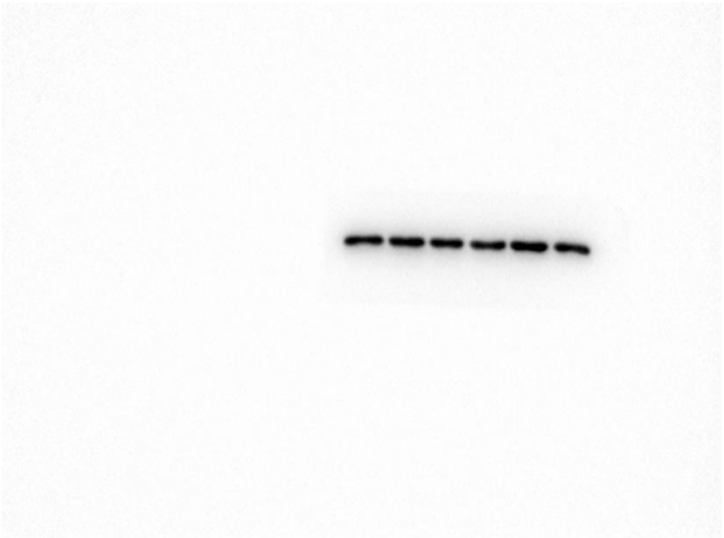

Figure 7

CD38

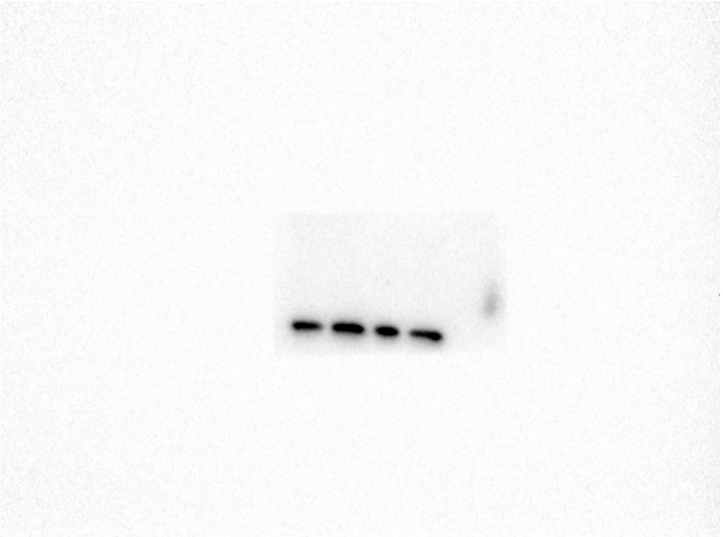

GAPDH

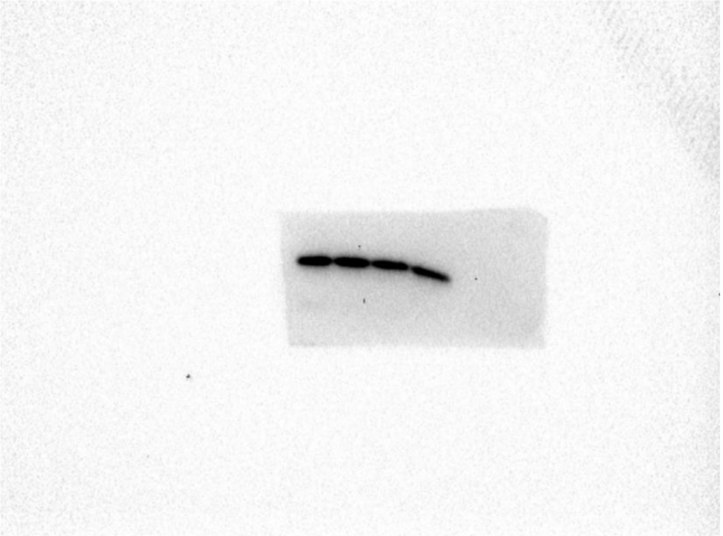

Supplement: Supplementary file 2 [file Data_Sheet_1.PDF]
